# Computational prediction and conformation of relationships among microbes, drugs and diseases

**DOI:** 10.1038/s41598-025-29306-6

**Published:** 2025-11-29

**Authors:** Hassan Shokri Garjan, Parvin Samadi Pakchin, Reza Ferdousi

**Affiliations:** 1 Department of Health Information Technology, School of Management and Medical Informatics, Tabriz University of Medical Sciences, Tabriz, Iran; 2 Research Center for Pharmaceutical Nanotechnology, Biomedicine Institute, Tabriz University of Medical Sciences, Tabriz, Iran

**Keywords:** Microbe-disease associations, Drug-disease relationships, Computational models, Computational prediction, Microbe-drug-disease, Computational biology and bioinformatics, Drug discovery, Microbiology, Diseases, Signs and symptoms

## Abstract

Complex and diverse microbial communities are closely linked to human health, and their study plays a vital role in advancing medicine, particularly personalized healthcare. Identifying potential microbe–disease–drug relationships is useful for drug discovery and clinical treatment, and it also improves our understanding of microbial mechanisms. Due to the complexity and cost of biological experiments, computational methods provide a rapid and efficient way to predict potential interactions between microbes, drugs, and diseases. In this article, we predict relationships between microbes, drugs, and diseases using existing similarity and interaction data through Cytoscape software. Some of the potential relationships were confirmed by the available information, while the others require further clinical investigation. Due to the critical role of the microbiome in disease and medicine, more research and information are needed in this field. In the future, the various interactions between drugs, microbes, and diseases may improve the understanding of personalized medicine, promote early diagnosis, and provide potential treatments for a wide range of diseases.

**Supplementary Information:**

The online version contains supplementary material available at 10.1038/s41598-025-29306-6.

## Introduction

The human microbiome, often referred to as the ‘last organ,’ consists of billions of bacteria inhabiting various ecological niches throughout the human body. The microbiome consists of living organisms such as bacteria, fungi, and archaea that make up microbiota (the term “gut microbiota” refers to the assortment of bacteria, archaea, and eukarya that colonize the gastrointestinal tract), as well as nonliving components such as phages, viruses, and plasmids^1^. These microbes form a dynamic and complex genetic pool and constantly interact with human cells and one another^2^. The microbiome consists of many more components than human cells, encoding 150 times more diverse genes than their human hosts^3,4^. The human gut microbiome (HGM) includes the majority of bacteria found in the lower gastrointestinal tract^5^.

Recent microbiome research has led to important medical breakthroughs. Historically, microbes have been regarded as harmful, and infectious diseases remain a major global health challenge. In recent years, the importance of the microbiome to human health and the need to maintain microbial diversity have been recognized^6–8^. An imbalance in the gut microbial community is referred to as dysbiosis HGM has been associated with a variety of diseases, including metabolic syndrome, immunological dysfunction, inflammatory bowel disease, and neurological problems, although the mechanisms are variably well understood^9,10^. In general, the metabolic processes of the microbiome facilitate physiological processes that are important for human health. Microbial enzymes, which exhibit a high degree of functional redundancy, convert a wide range of chemical substrates^12^. Thanks to culture-independent ‘omics’ approaches, the past 15 years have brought significant advances in identifying microbial genes and their products^13^. More than 10 million non-redundant microbial genes (compared to 30,000 human genes) have been found from over 1200 human fecal microbiomes collected in Europe, the USA, and China, which has led to the widespread belief that the metabolic potential of gut microbiota far exceeds that of the human host^14^. Although many bacterial gene functions remain unknown, the gut microbiota has been shown to influence the host in several important ways. It helps protect against pathogens, modulates immune responses^11,12^, metabolizes bile acids and xenobiotics^13,14^, and contributes to intestinal, neurological, and skeletal health^15–18^. The gut microbiota also plays a key role in regulating host metabolism^19–25^. In addition, the influences of the microbiota on the physiological activity of drugs are just becoming clear^26^.

Most drug-microbiome interactions have only recently been characterized, due to advances in genomic, metabolomic, and microbiological approaches^27^. More than 180 drugs have already been identified as substrates for bacterial enzymes in the gut, making them susceptible to enzymatic change in vivo^28,29^. It’s becoming obvious that microbial metabolism can have a major impact on the effects of drugs in the body. Each person’s microbiome composition is unique, much like a fingerprint^30^. Therefore, differences in an individual’s physiological and clinical response to drug therapy could be influenced by the composition of their microbiome^31^. Moreover, the interaction between drugs and the microbiome is bidirectional: just as the microbiota can have an impact on drugs, drug administration can also affect the microbiome^32^. Over the past decade, our understanding of how the human gut microbiota contributes to health and disease has improved dramatically. We now better understand how it changes across life stages, regions, and environmental conditions (The Integrative Human Microbiome Project (HMP (iHMP)) Research Network Consortium, 2019)^33–35^. The gut microbiome, particularly at the bacterial strain level, is currently thought to have a very unique composition^30^. Moreover, most healthy individuals maintain a consistent microbiota composition throughout adulthood, which is shaped early in life and influenced more by environment than genetics^36–40^. Conversely, the therapeutic efficacy of drugs can be affected by their chemical modification through intestinal bacteria. Severe disturbances like dietary changes or indiscriminate antibiotic use may disrupt microbiome stability, and its potential for recovery remains uncertain^35,41,42^. Due to the complexity and cost of biological experiments, computational methods can efficiently predict potential relationships between biological components and serve as a useful complement^43–45^.

Researchers have developed various computational methods to infer potential microbe–disease^46^, Drug–Disease^47–49^, and Microbe–Drug^50–52^Associations based on the vast amount of biomedical data that has been accumulated as a result of the rapid development of techniques in genomics, proteomics, life sciences, and pharmaceutical research. Drug administration can have a substantial impact on clinical drug response due to its specific effects on the microbiome and metabolic processes of the human body. This suggests that microorganisms, drugs, and diseases are intricately linked. Microorganisms, medications, and diseases are intricately connected, as the microbiome regulates many essential physiological processes, drug administration has particular effects on the microbiome, and microbiological metabolism can have a substantial impact on clinical drug response. In order to better understand underlying disease mechanisms from the viewpoint of human microbes and drugs, it may be crucial to identify potential pairwise associations between microbes, drugs, and diseases. This information could greatly aid pathogenesis research, support early diagnosis, and contribute to the development of precision medicine. However, standard clinical trials for identifying connections between medications, diseases, and microbes are both costly and time-consuming. This study introduces a novel tripartite relationship model between drugs, microbes, and diseases. This model improves our understanding of drug–microbe and disease–microbe interactions and provides a unified framework connecting all three. In this study, data from current databases, clinical publications, and experimental trials were used to predict whether there is a relationship between diseases and microbes and between microbes, and drugs established correlations between microbes and microbe-disease and microbe-drug were investigated.

**Fig. 1:**
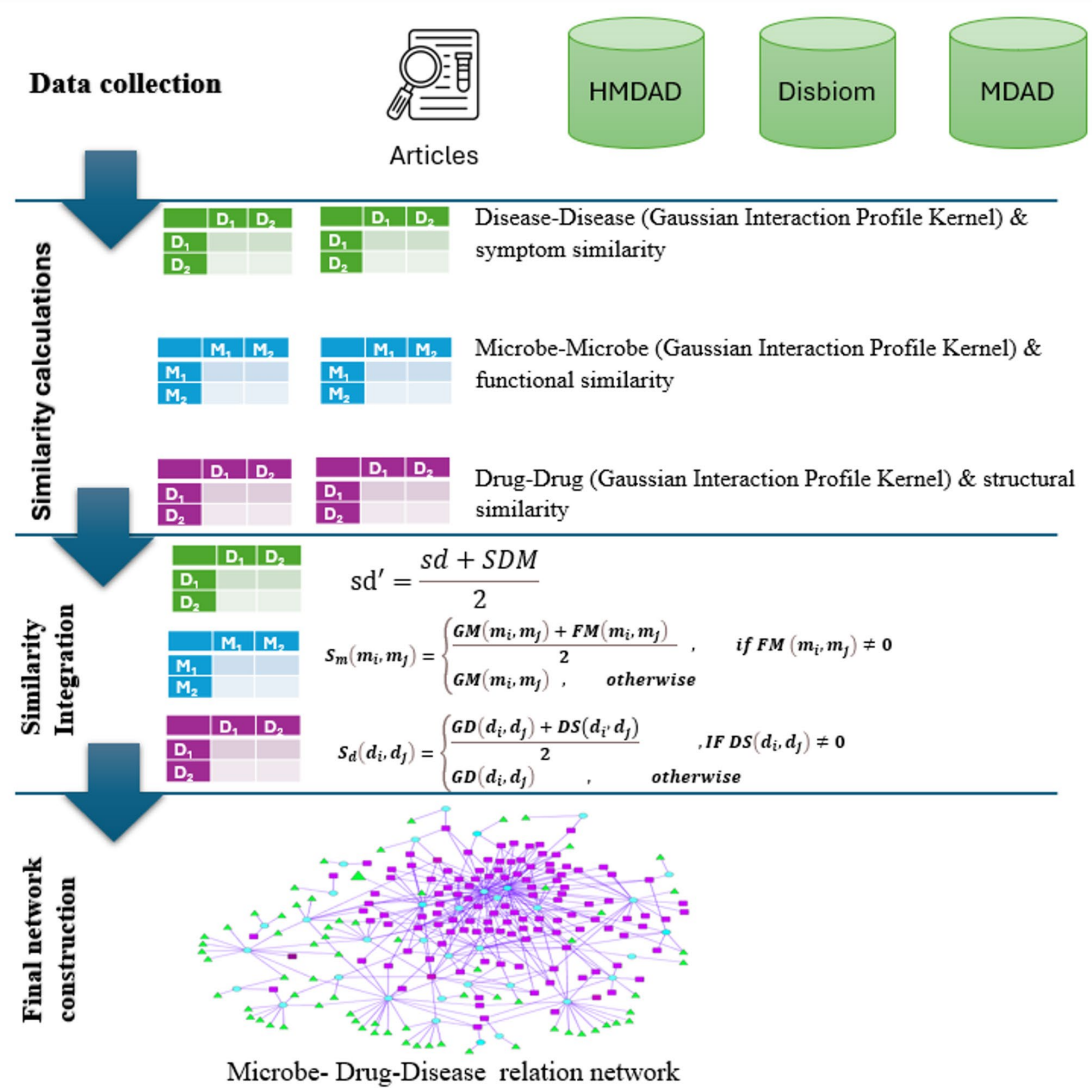
The general flowchart of the method based on the known Disease-Microbe-Drug Society network.

The methodology adopted in this study is summarized in Fig. 1.The figure begins by illustrating the collection of data from databases and experimental studies. It then demonstrates the construction of similarity matrices, the measurement of component similarity, and the use of Cytoscape software to create three-way connections. To enhance prediction power and biological interpretability, the model employs Random Walk with Restart (RWR) algorithms on the integrated similarity network, allowing the propagation of association signals across the tripartite structure. Unlike deep learning models that often operate as “black boxes,” this method offers high transparency and traceability in prediction logic.

## Materials and methods

### Dataset

We got information about known links between microbes and diseases from the Human Microbe-Disease Association Database (HMDAD) and Disbiome databases. HMDAD is a resource that collects and organizes data from microbiota research showing connections between human microbes and diseases.This database contains 483 empirically confirmed microbe–disease associations involving 39 diseases and 292 microbes^53^.

Disbiome is a curated database that documents changes in microbial composition associated with various diseases. Users can search by disease, detection method, or organism to retrieve detailed experimental data, including disease–bacteria associations, abundance comparisons, control types, detection methods, and references. Disbiome has compiled 5,573 empirically validated human microbe–disease associations from previously published literature and databases, covering 240 diseases and 1,098 microbes. These data were sourced from two databases, comprising a total of 4,802 correlations between 257 diseases and 1,334 microbes^54^.

The Microbe–Drug Association Database (MDAD) was used as a source for known microbe–drug associations^55^. The MDAD compiles relationships between microbes and medications from multiple drug databases and publications, all supported by clinical or experimental evidence. It offers comprehensive annotations for each entry, including original references, UniProt data on microbial targets, and molecular drug information from DrugBank.

The MDAD dataset contains 5,505 clinically or experimentally validated microbe–drug associations involving 1,388 drugs and 174 microbes. After removing redundant entries, 2,470 unique associations involving 1,373 drugs and 173 microbes were retained.

### Construction of similarity networks

To construct a heterogeneous network, disease–disease, drug–drug, and microbe–microbe similarity networks were built independently. The similarity of each pair—whether microbe–microbe, disease–disease, or drug–drug—was calculated to support this structure. In this study, Gaussian interaction profile kernels and biological similarity metrics were employed to evaluate similarities between microbes and diseases.

#### Gaussian interaction profile kernel similarity

##### Similarity for microbes

We computed inferred microbe similarity based on the topological structure of the known microbe–disease association network using Gaussian kernel interaction profiles. This approach considers the assumption that microbes associated with more prevalent human diseases are more likely to share higher functional similarity. The association between a microbe m_i_ and all diseases under investigation is represented by its interaction profile, denoted as IP(m_i_), which corresponds to the i‑th row of the association matrix Y. Based on their interaction profiles, IP(m_i_) and IP(mⱼ), the inferred similarity between any two microbes m_i_ and mⱼ can be computed as follows:

1 $$\:GM\left({m}_{i},{m}_{j}\right)=\text{exp}\left({-\eta\:}_{d}\right.||IP\left({m}_{i}\right)-IP\left({m}_{j}\right){\Vert\:}^{2})\:\:$$

where the kernel bandwidth is controlled by the parameter $\:{\eta\:}_{\varvec{d}}$. The parameter $\:{\eta\:}_{\varvec{d}}$is updated using a normalized bandwidth calculated from the average number of associations per microorganism.

2 $$\:{\eta\:}{d}=\acute{\eta}{d}/\left(\right(\frac{1}{nd})*\sum\:{i=1}^{nd}\left.||IP\left({m}{i}\right){\Vert\:}^{2}\right)$$

In this case, the prior study assigned $\acute{\eta}_{d}$ to 1 for the simplified computation^56^. In this manner, the inferred microbe similarity might be represented by a GM matrix, where GM(i, j) indicates the probable similarity between microbes mi and m_**j**_.

##### Similarity for diseases

The Gaussian interaction profile kernel similarity for diseases (GD) was computed in the same manner as that for microbes.

3 $$\:GD\left({d}_{i},{d}_{j}\right)=\text{exp}\left({-\eta\:}_{d}\right.||IP\left({d}_{i}\right)-IP\left({d}_{j}\right){\Vert\:}^{2})$$

4 $$\:{\eta\:}_{d}=\acute{\eta}_{d}/(\frac{1}{nd}\sum\:_{i=1}^{nd}\left.||IP\left({d}_{i}\right){\Vert\:}^{2}\right)\:\:\:\:\:\:\:\:$$

where $\acute{\eta}_{d}$ was set to 1 as well.

#### Biological information

To enhance biological relevance, multiple similarity measures were integrated, including Gaussian interaction profile kernels, disease symptom similarity, microbial functional similarity, and drug structural similarity, as described below.

##### Function similarity microbe

The functional similarity matrix of the microbe (FM) was calculated using the method proposed by Kamenova (2017). By adding the gene families, or network nodes, that are present in at least one of the genomes under study, and the linkages that connect those gene families, or network edges, to the reference network, a protein-protein functional association network for a specific pair of species is created. Gene families are labeled based on whether their proteins are found in genome A, genome B, or both—classified as A-only, B-only, or shared (present in both). The six possible undirected edges between gene family types are A–A, B–B, shared–shared, A–shared, B–shared, and A–B. The index of similarity for genome content is calculated as follows. In gene set 1, four gene families are either present or absent in both genomes A and B, yielding a similarity score of 2 based on matched presence/absence. Six gene families make up gene set 1, meaning that on average, 0.33 of them have the same presence/absence status.

The functional connectivity index between two microbes is calculated as follows. In this case, three gene families are encoded exclusively in genome A, two solely in genome B, and three are shared by both genomes. The genomes of species A and B encode genes from five and six gene families, respectively.

The edges connecting gene families are classified into six types, depending on the genome origin of the connected nodes. Edges linking gene families exclusive to genomes A and B must span organismal boundaries to exist in a two-species network. These A–B edges, representing connections between proteins encoded in different genomes, require cross-genomic interactions. In contrast, more edges can exist within the same genome due to denser intra-genomic connectivity. Therefore, the functional connectivity index between two microbes is defined as the proportion of cross-genomic edges (i.e., those connecting gene families exclusive to different genomes) among all possible edges linking genome-specific gene families^57^. In this method, a functional protein–protein association network is required, in which nodes represent gene families from each genome, and edges correspond to gene interaction scores obtained from the STRING database (https://string-db.org). This network framework enables the accurate calculation of functional similarity between microbes from different species.

##### Symptom-Based disease similarity (SDM)

Feature vectors are commonly used to represent text documents or concepts in the field of information retrieval. Each disease j is represented by a symptom-based feature vector d_**j**_.

5 $$\:{d}_{j}=\left({w}_{1,j},{w}_{2,j},\dots\:..,{w}_{n,j}\right)\:$$

where w_**i, j**_ is a measure of how strong the association between symptom i and disease j is. The frequency of symptom occurrence across different diseases varies significantly. To account for this heterogeneity, word frequency and the inverse of document frequency w_**i, j**_ were used instead of absolute co-occurrence w_**i, j**_ to assess the strength of the association between symptom i and disease j.

6 $$\:{w}_{i,j}={w}_{i,j}log\frac{N}{{n}_{i}}\:\:$$

Here, N denotes the total number of diseases in the dataset, and n_**i**_ represents the number of diseases associated with symptom i. Thus, the similarity between the two disease vectors d_**x**_ and d_**y**_ is determined as follows:

7 $$\:\text{cos}\left({d}_{x},{d}_{y}\right)=\frac{{\sum\:}_{i}{d}_{x,y},{d}_{y,i}}{\sqrt{{\sum\:}_{i}{{d}_{x,i}}^{2}}\:\:\:\:\:\:\:\sqrt{{\sum\:}_{i}{{d}_{y,i}}^{2}}}\:\:\:$$

Cosine similarity ranges from 0 (indicating no shared symptoms) to 1 (indicating identical symptom profiles)^58^.

##### Drug structural similarity matrix

We employed the SIMCOMP technique to construct the structural similarity matrix for drugs (DS). Each entry in the matrix provides atom alignment data between the query compound and database molecules. The similarity between the query and a database entry is determined by the ratio of matched atoms to the total number of matched and unmatched atoms from the atom alignment. To define the similarity between chemical graphs, we used the Tanimoto coefficient (with a threshold of 0.8) between bit-represented vectors, as it is widely recognized in chemical structure search systems^59^.

#### Integration of Gaussian kernel matrices and biological information

To balance topological similarity captured by the Gaussian kernel with biological or functional similarity, we integrated the two using simple averaging. The Gaussian kernel acts as a soft-weighted measure of closeness, where nearby data points contribute more strongly and distant ones less. In parallel, averaging biological similarities (e.g., across pathways or cellular responses) reduces random fluctuations and highlights stable patterns. Thus, averaging both sources provides a robust composite score that reduces noise and emphasizes consistent relationships across computational and biological domains.

To improve microbial similarity representation, we constructed a new network by integrating functional similarity with Gaussian kernel-based microbial similarity. In particular, for m_**i**_ and m_**j**_ microbes, if there is a functional microbial similarity between them, the integrated microbial similarity is defined as the average of GD and DS; GD otherwise The integrated microbe similarity of S_**m**_ is defined as follows^60^:

8 $$\:{S}_{m}\left({m}_{i},{m}_{j}\right)=\left\{\begin{array}{c}\frac{GM\left({m}_{i},{m}_{j}\right)+FM\left({m}_{i},{m}_{j}\right)\:\:}{2},\:\:\:if\:FM\:\left({m}_{i},{m}_{j}\right)\ne\:0\:\:\\\:GM\left({m}_{i},{m}_{j}\right),\:\:\:otherwise\end{array}\right.$$

A final drug similarity is generated by combining the similarity of the drug structure and the similarity of the Gaussian kernels to complement the biological information and improve the drug similarity. If a structural match exists between drugs d_**i**_ and d_**j**_, their similarity is computed as the average of GD and DS; otherwise, GD is used alone. In this manner, the integrated drug similarity S_**d**_ is defined:

9 $$\:{S}_{d}\left({d}_{i},{d}_{j}\right)=\left\{\begin{array}{c}\frac{GD\left({d}_{i},{d}_{j}\right)+DS({d}_{i},{d}_{j})}{2},\:IF\:DS\left({d}_{i},{d}_{j}\right)\ne\:0\:\:\\\:GD\left({d}_{i},{d}_{j}\right),\:\:\:\:\:\:\:\:otherwise\end{array}\right.$$

As previously mentioned, the matrices for drug and microbe similarity are S_**d**_ and Sm, respectively. Each row or column in S_**d**_ (or S_**m**_) represents a drug’s (or microbe’s) similarity profile, which may be thought of as the drug’s (or microbe’s) feature vector. However, as the computed similarity may contain some noise from false positives and computational limits, it is not enough to just consider the similarity profiles as input characteristics for bacteria and medications. To extract features from similarity profiles, we applied a restart-based random walk technique. Random walk with restart is a network-based technique that captures both local and global topological features. Random walk with restart is defined formally as follows^61^:

10 $$\:{p}_{i}^{t+1}=\left(1-\varphi\:\right)M{p}_{i}^{t}+{\varphi\:e}_{i}\:\:$$

where M (i.e., S_d_ or S_m_) represents the transition probability matrix and φ is the restart probability, which is empirically set as 0.9. In addition, e_i_ ∈ R^*n*×1^ is the initial probability vector for the i-th node, and e_ij_ is 1 if j = i and 0 otherwise. $\:{p}_{i}^{t}$ ∈ R^*n*×1^ shows the probabilities of reaching other nodes at the time t from the i-th node, and we take $\:{p}_{i}^{t}$ at steady state as the feature vector for the i-th node. We performed random walk with restart on both drug and microbe similarity networks to generate a probability profile vector for each. This probability profile vector can thus form a new drug feature matrix F^d^ ∈ R^nd×nd^ and a new microbe feature matrix F_m_ ∈ R^nm×nm^. To ensure comparability, we normalized each probability profile vector in F_d_ and F_m_ so that their elements sum to 1. The normalized vectors from F_d_ and F_m_ are then used as input features for microbes and drugs in the model. Consistent with the heterogeneous network, a new feature matrix X ∈ R^(nd+nm)×(nd+nm)^ is described as follows:

$$\:X=\left[\begin{array}{cc}0&\:{F}_{d}\\\:{F}_{m}&\:0\end{array}\right]$$

Microorganism and disease similarity is measured using Gaussian interaction profile kernel similarity. However, since the Gaussian interaction profile of microbial similarity is a measurement based on association information, other types of microbe or disease similarity based on different biological data must also be included. Indeed, several researchers have proposed methods to assess microbe or disease similarity based on diverse biological characteristics^62^.

Several studies have introduced a symptom-based human disease network (HSDN) to assess disease similarity through co-occurrence of disease–symptom keywords. In this study, the HSDN was used to calculate symptom-based disease similarity (SDM), and then a new disease similarity (sd) matrix was constructed by averaging SDM and Sd^′^ in a way^63^.

11 $$\:\text{s}\text{d}^{\prime}=\frac{sd+SDM}{2}$$

## Result

**Fig. 2:**
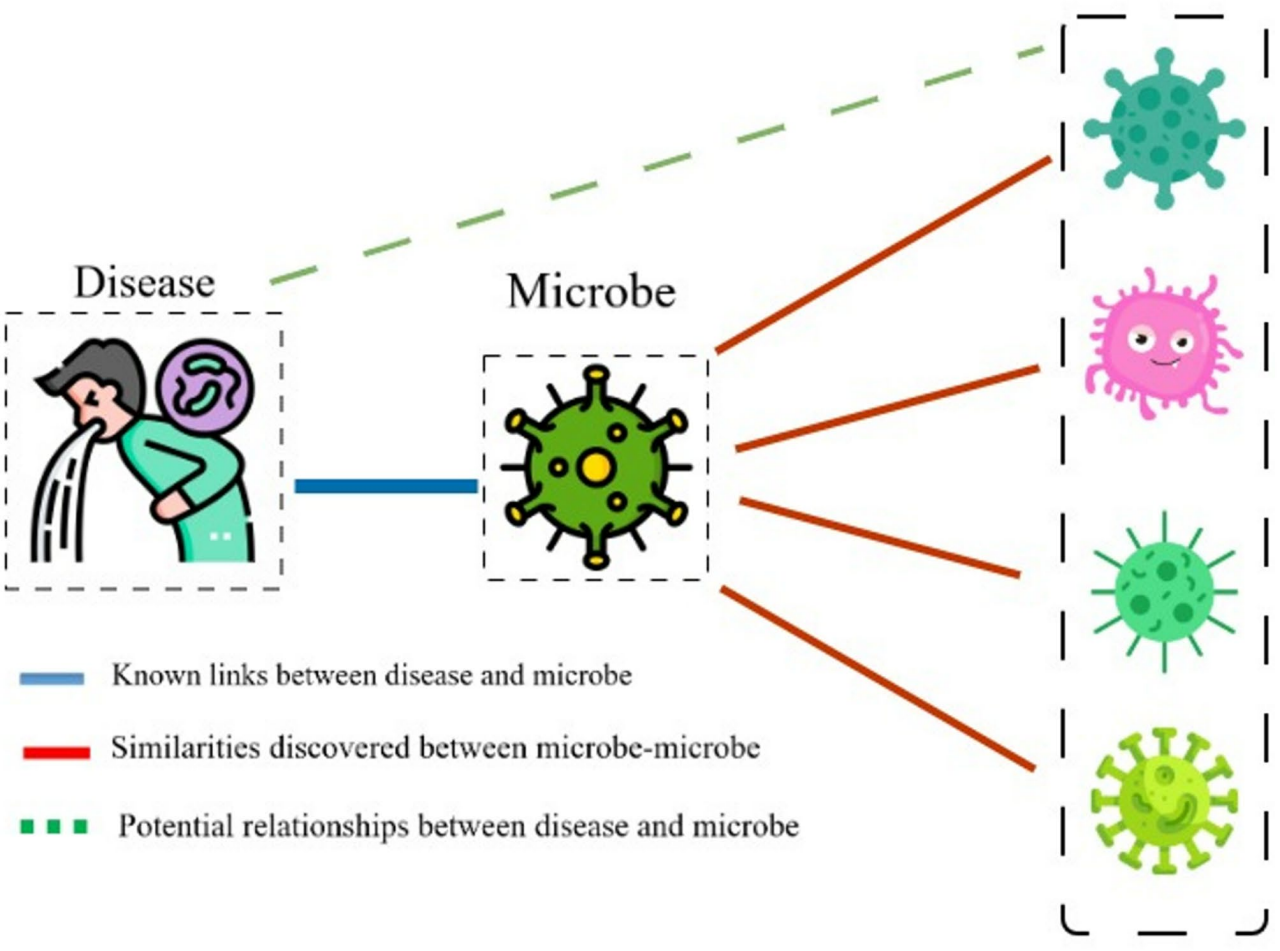
The relationship between disease, microbe-microbe (The icons used in this figure were obtained from Flaticon.com under a free license on November 6, 2025, by Hassan Shokri Garjan).

Pairwise similarities among diseases, drugs, and microbiomes were calculated using established similarity measures. The gene-based human disease network (HGDN)^53^ and the symptom-based human disease network (HSDN)^64^ were obtained from previous studies. The functional similarity matrix of microbes was calculated using the method proposed by Kamenova (2017)^57^. In addition, the structural similarity matrix of the drug was constructed using the SIMCOMP method^59^. Figure 2 illustrates how microbiome–disease relationships and microbe–microbe similarities help identify additional disease–microbiome associations.

**Fig. 3:**
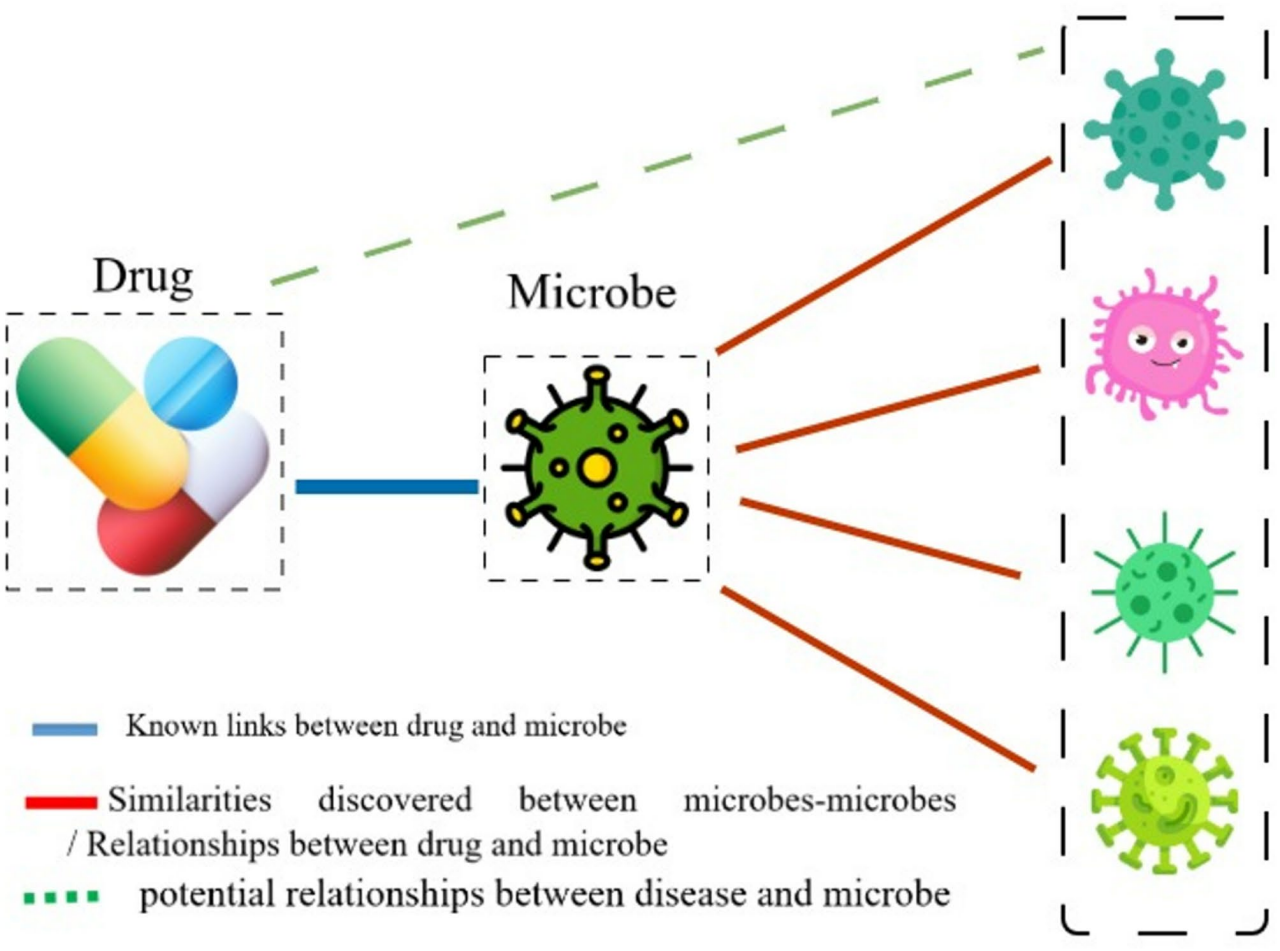
Drug-microbe-microbe relationship (The icons used in this figure were obtained from Flaticon.com under a free license on November 6, 2025, by Hassan Shokri Garjan).

The similarities between microbe-microbe, disease-disease, and drug-drug were calculated. Additionally, known microbe–disease and microbe–drug associations were retrieved from clinical laboratory databases. Based on existing similarities and known associations, if microbe X is similar to microbes X_1_ through X_n_, and X is linked to disease (or drug) L, it can be inferred that X_1_ through X_n_ may also be associated with L.Therefore, it can be predicted that the microbes X_1_, X_2_,…, X_n_ are also associated with disease L. To explore overlapping relationships that could indicate novel disease–microbe–drug associations, several case studies were analyzed. Figure 3 illustrates how microbe–drug relationships and microbe–microbe similarities help identify additional drug–microbe associations.

**Case study 1**: **Type 1 diabetes is associated with a decrease in Acetanaerobacterium elongatum microbiome**.

The intestinal microbiota of children with at least two diabetes-related autoantibodies (*n* = 18) was compared. A deficiency of lactate- and butyrate-producing species was linked to T-cell autoimmunity. Principal component analysis revealed a correlation between low abundance of these species and reduced insulin sensitivity. The most prominent phyla were Firmicutes (58.1%), Actinobacteria (36.2%), and Bacteroidetes (3.4%), as revealed by a phylum-level analysis of the high-level readings of all samples. The most abundant families were Bifidobacteriaceae (32.8%; Actinobacteria), Lachnospiraceae (18.4%; Firmicutes), and Ruminococcaceae (17.1%; Firmicutes). The Bifidobacterium genus was the most common at the genus level (34.2%). The Bacteroidetes phylum (4.6%), the Bacteroidaceae family (2.5%), and the Bacteroides genus (3.1%) were all more abundant in autoantibody-positive than in autoantibody-negative children (4.6 vs. 2.2%, 3.5 vs. 1.5%, and 4.3 vs. 2.0%, respectively; *P* = 0.035, 0.022, and 0.031, respectively; Mann-Whitney U test)^65^. Table 1 lists several examples of microbes similar to Acetanaerobacterium elongatum obtained by similarity. Their associations with type 1 diabetes (T1D) were examined using database information and literature evidence^65^.

**Case study 2: The relationship between Bifidobacterium and T1D is reduced. The same was observed in the other cases**.

Those with type 1 diabetes (T1D) are more likely to experience micro- and macrovascular problems. Several studies have shown that the gut microbial makeup of people with T1D differs from that of healthy people. Inflammation, changes in intestinal permeability, and abnormalities in metabolites might result from these changes in the gut ecology. Together, these impacts may have an influence on the metabolic regulatory system, which in turn may affect blood glucose regulation.According to this review, the abundance of Dorea formicigenerans, Bacteroidetes, Lactobacillales, and Bacteroides was favorably connected with the HbA1c level in T1D patients, whereas the abundance of Prevotella, Faecalibacterium, and Ruminococcaceae was negatively connected. Rather, there was a negative correlation between fasting blood glucose and Bifidobacteria. Furthermore, Clostridiaceae and time in range were positively correlated. Additionally, it was shown that gut dysbiosis and inflammatory parameters were positively correlated in T1D patients.

**Table 1:** Relationships between disease and microbe along with empirical sources.

Disease	Microbe	Ref	Result
T1D	Bifidobacterium	^66,67^	Type 1 diabetes patients often experience micro- and macrovascular issues due to changes in gut microbial makeup. These changes may lead to inflammation, intestinal permeability, and metabolite abnormalities, impacting the metabolic regulatory system and blood glucose regulation. The abundance of certain bacteria, such as Dorea formicigenerans, Bacteroidetes, Lactobacillales, and Bacteroides, is positively correlated with HbA1c levels.
T1D	Clostridium butyricum	^68^	To assess the effects of CB0313.1 therapy, pyrosequencing of the gut microbiota and flow cytometry analysis of immune cells in the mesenteric lymph node (MLN), pancreatic lymph node (PLN), pancreas, and spleen were conducted. Early oral administration of CB0313.1 resulted in reduced insulitis, delayed onset of diabetes, and improved energy metabolic dysfunction. Additionally, 16 S rRNA gene sequencing revealed that CB0313.1 enriched butyrate-producing bacterial subgroups, increased the Firmicutes/Bacteroidetes ratio, and enhanced Clostridium subgroups.
T1D	Lactococcus lactis	^69^	Additionally, L. lactis-producing SNase had protective effects on pancreatic islets and reduced small intestinal inflammation in NOD mice, according to the results of HE staining. Therefore, the results of the present investigation suggest that oral SNase administration by L. lactis can successfully prevent T1D, reduce inflammation, and support immunomodulatory balance in NOD mice.
T1D	Succiniclasticum	^70^	In contrast to fresh-onset patients, seronegative FDRs had a higher concentration of the Bacteroidetes species Alistipes (adjusted *p* = 0.02). In seronegative FDRs compared to new-onset patients, there was a reduction in the number of the Firmicutes genera Lactobacillus and Succiniclasticum as well as the Bacteroidetes genera Prevotellaceae (adjusted *p* = 0.07 for Prevotellaceae and Lactobacillus and *p* = 0.01 for Succiniclasticum).

The results between Sorivudine and Bacteroides are as follows.

Sorivudine (1-beta-D-arabinofuranosyl-5-(E)-(2-bromovinyl)uracil) is an effective antiviral agent targeting varicella-zoster virus and herpes simplex virus type 1. However, caution is advised when using sorivudine in combination with anticancer drugs such as 5-fluorouracil (5-FU). This is due to the fact that (E)-5-(2-bromovinyl)uracil (BVU), a metabolite of sorivudine, inhibits the degradation of 5-FU, leading to increased accumulation in the blood and heightened toxicity. Since phosphorolytic enzymes generate BVU from sorivudine, their activity distribution was analyzed in rats. High activity was observed in the cecum and colon, whereas very low or no activity was detected in the liver, kidney, stomach, cecum, colon, gastric, and small intestinal contents. These findings suggest a significant role of intestinal microflora in BVU production. Consequently, phosphorylase activity was measured in cell-free extracts of 23 aerobes, 16 anaerobic bacteria, and a fungus. Among them, Bacteroides species B. vulgatus, B. thetaiotaomicron, B. fragilis, B. uniformis, and B. eggerthii were identified as dominant members of the intestinal microflora, showing high activity in converting sorivudine to BVU. To elucidate the contribution of the intestinal microflora to BVU production in vivo, sorivudine was administered to rats treated with various antibiotics, and the concentration of BVU in the serum of the rats was measured. When sorivudine was administered to rats treated with ampicillin or a combination of bacitracin, neomycin, and streptomycin, only a small amount of BVU was detected in the serum, attributed to the decreased numbers of viable aerobes and anaerobes. Furthermore, the serum BVU concentration in rats treated with metronidazole to reduce the number of anaerobes in the gut was also significantly low. Conversely, the serum BVU concentration in rats treated with kanamycin, selectively used to reduce the number of aerobes, was higher than that in non-treated rats. These findings support that the BVU is primarily produced by intestinal anaerobic bacteria, especially Bacteroides species, in vivo^71^.

Based on the similarities, several examples of microbes similar to Bacteroides were identified and listed in Table 2. The association of these microbes with sorivudine was investigated using database information and articles, and evidence for them was provided. (Supplementary file). The same was observed for the other cases )drugs).

Sorivudine (1-β-D-arabinofuranosyl-5-(E-2-bromovinyl)uracil) is a potent and specific antiviral medication that targets the varicella-zoster virus and herpes simplex virus type 1. However, severe adverse effects (such as bone marrow suppression) have been reported when sorivudine is used in combination with anticancer medications related to fluorouracil (5-FU). Researchers have investigated the mechanism underlying the fatal toxicity of this combination treatment. In mice, the plasma half-life of 5-FU and its area under the curve were significantly increased when sorivudine or its primary metabolite, 5-(E-2-bromovinyl)uracil (BVU), was administered one hour prior to 5-FU injection.

While a single dose of sorivudine, BVU, 5-FU, or tegafur did not cause any infection, the combination of sorivudine (100 mg/kg), BVU (20 mg/kg), and sublethal doses of 5-FU (no more than 160 mg/kg) or tegafur (500 mg/kg) led to a lethal endogenous infection by *Escherichia coli* in all treated mice. The administration of streptomycin sulfate protected the mice from the lethal toxicity of these drug combinations, suggesting that bacterial infection is the underlying cause of the toxicity. Furthermore, parenteral administration of a heat-killed preparation of *Lactobacillus casei* YIT 9018 (LC 9018) successfully prevented the lethal endogenous infection and accelerated recovery from chemotherapy-induced hematopoietic injury. These findings suggest that nonspecific immunopotentiation plays a crucial role in mitigating the severe side effects of sorivudine when used alongside anticancer drugs related to 5-FU^72,73^.

**Table 2:** The empirical studies on relationships between sorivudine and microbe.

Drug	Microbe	Ref	Result
Sorivudine	Bacteroides uniformis	^71^	High levels of 5-(E)-(2-bromovinyl)uracil (BVU), produced by the metabolism of sorivudine by intestinal anaerobic bacteria, significantly increase both the concentration and toxicity of 5-fluorouracil (5-FU). The predominant Bacteroides species in the intestinal microflora—B. vulgatus, B. thetaiotaomicron, B. fragilis, B. uniformis, and B. eggerthii—have demonstrated high conversion rates of sorivudine to BVU.
Sorivudine	Bacteroides fragilis	^71^	Elevated blood levels of 5-(E)-(2-bromovinyl)uracil (BVU), resulting from the hydrolysis of sorivudine by intestinal anaerobic bacteria, increase both the systemic exposure to and toxicity of 5-fluorouracil (5-FU). The predominant Bacteroides species in the intestinal microflora—B. vulgatus, B. thetaiotaomicron, B. fragilis, B. uniformis, and B. eggerthii—have demonstrated significant capacity to convert sorivudine into BVU.
Sorivudine	Varicella-zoster virus	^72–74^	Both the time to crusting of varicella lesions (from 6.6 to 5.8 days; *P* = 0.004) and the number of days to new lesions (from 3.9 to 3.1 days; *P* = 0.014) were significantly shortened in the sorivudine group in this double-blind, controlled study of 40 mg of sorivudine daily for 5 days compared with placebo. Additionally, the time it took for the virus to shed from the skin decreased (from 3.3 to 2.6 days; *P* = 0.002). One of the earliest pieces of clinical data introducing the antiviral drug sorivudine as a successful therapy for varicella-zoster virus infections was this publication.
Sorivudine	Streptococcus mitis	^75^	Sorivudine has been evaluated in 14-day dose-ranging trials at daily doses of 10, 20, 40, 80, and 100 mg. Plasma levels of 0.5 µg/ml were achieved at doses below 10 mg/day, whereas the peak plasma concentration at the highest dose tested (100 mg) was 5.0 µg/ml. In U.S.-based studies assessing the efficacy and safety of sorivudine, a dose of 40 mg once daily was used. This dose yielded average plasma concentrations of 1.8 µg/ml and peak concentrations of 0.2 µg/ml. The average plasma elimination half-life is approximately 5 to 7 h. Notably, even at a dose as low as 10 mg/day, the plasma concentration of sorivudine exceeds the effective dose (ED) threshold for varicella-zoster virus (VZV) by a factor of 10. These findings highlight the strong therapeutic potential of sorivudine for the treatment of VZV infections.

### Discovery of the triple relation disease-microbe-drug

Numerous clinically and computationally confirmed examples have shown how microorganisms function as bridges between medications and illnesses, supporting the growing body of research into microbiome-related therapies. We examine both case studies supported by the literature and seminal clinical applications involving drug–microbe–disease triplets in order to situate the suggested paradigm within the present scientific environment. These demonstrate the suggested computational framework’s practical applicability and translational potential.

**Fig. 4:**
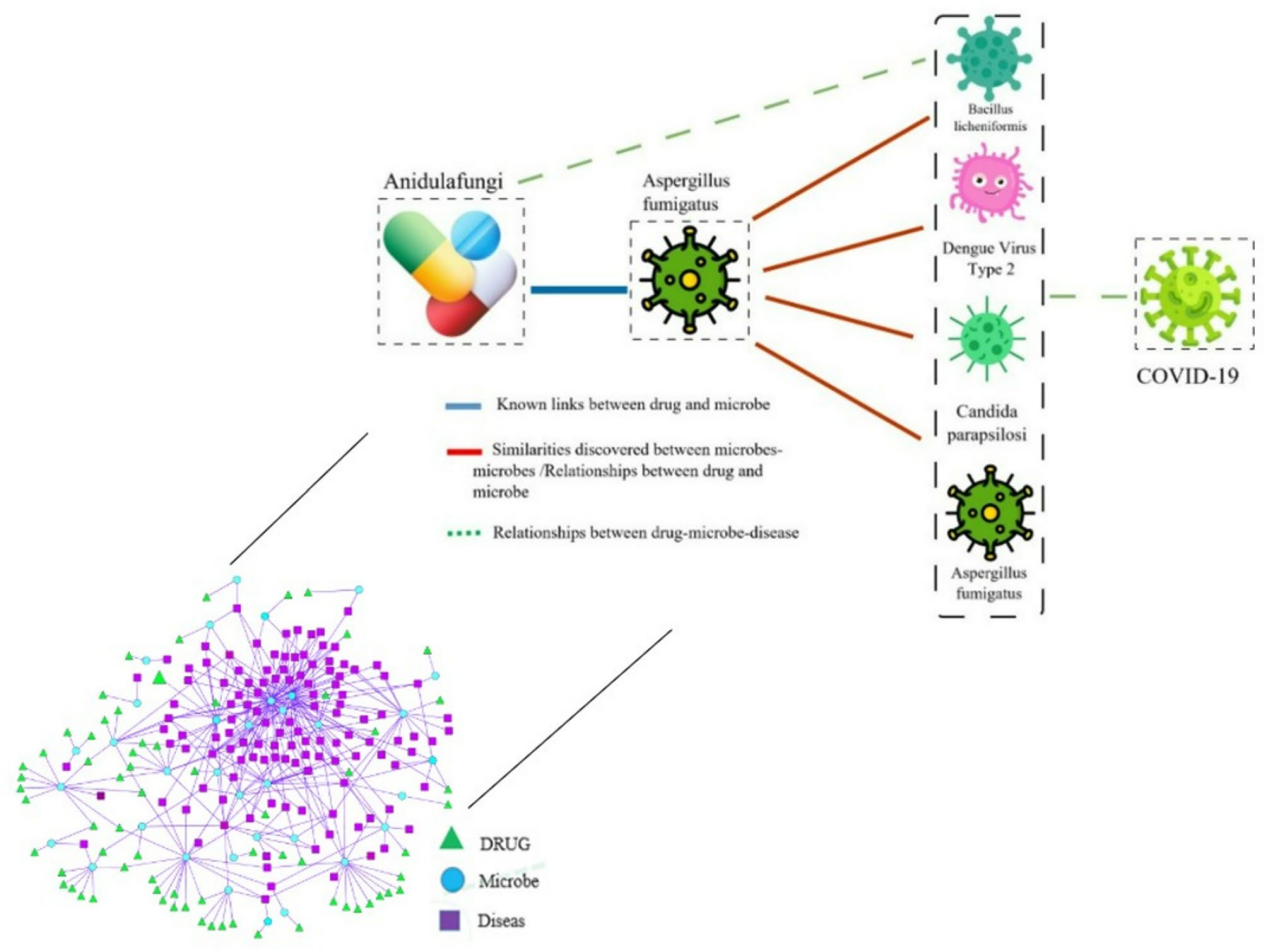
Multiple drug-microbe-disease interactions. The relationship between the AFG drug, the Aspergillus fumigatus microbiome, and the disease of COVID-19 has been drawn(The icons used in this figure were obtained from Flaticon.com under a free license on November 6, 2025, by Hassan Shokri Garjan).

To construct the disease–microbe–drug network, microbiomes associated with both diseases and drugs were identified and compared (see Supplementary file). Then, according to the assumption that because the disease (drug) separately causes the increase or decrease of microbes, the drug affects (improves or exacerbates) the disease by increasing or decreasing the microbiome. The results obtained from the triple relation disease-microbe-drug networks were examined and discussed. Figure 4 shows that more new relationships can be discovered from the discovered triple relationships of disease, microbiome, and drug. For example, the relationship between anidulafungin, Aspergillus fumigatus, and COVID-19 has been confirmed by experimental data and authoritative sources.

Based on the collected information on diseases and drugs associated with a common microbe, the links between microbes were identified in the previous sections. Their connections were drawn manually using Cytoscape software. These relationships were then studied in two ways. The first part of the study confirmed disease–drug associations mediated by a shared microbe, as supported by the literature. In addition, in the study of multiple relationships, the microbiome has different effects on the disease, and the use of drugs for different diseases also has different effects on the microbe. When the relationship of the components of the microbiome changes, the diseases improve or worsen, or there is drug resistance or even the occurrence of other diseases.

### Experimental validation

To calculate the performance of the work computationally, from all the similarities obtained, due to a large number of cases, 100 initial cases were investigated. 100 cases were examined for the disease-microbe, of which 86 cases were found as sources in laboratory and clinical articles. This means that about 86% approved the target prediction. While due to the less information and study about the drug-microbe relationship, 10 cases were found out of 100 cases, i.e., in about 10% of clinical and laboratory articles.

### Experimental comparisons

Based on the available literature, AFG demonstrated significant efficacy against 10 isolates of Aspergillus niger (A. niger) under laboratory conditions, assessed through both broth microdilution and disk diffusion methods. The effectiveness of AFG at doses of 1, 5, and 10 mg/kg was evaluated using a mouse model infected with A. niger comprising six isolates. AFG exhibited a capacity to reduce the mortality rate, with survival rates ranging from 70 to 100%, 60–100%, and 30–60% in mice treated with AFG at 10, 5, and 1 mg/kg, respectively. Additionally, AFG demonstrated a dose-response efficacy in diminishing tissue burden in the kidneys and spleen. Furthermore, a parallel experiment indicated that AFG administration did not result in a decrease in serum galactomannan concentrations in rats. Moreover, the histopathological studies also confirmed the effectiveness of AFG^76^. Using the mentioned methods, 96 cases of microbes were obtained that were similar to A. niger, and then it was checked whether AFG had an effect on them, and 8 cases confirmed the obtained results using experimental laboratory articles, or No results were found for similarities. Which requires more clinical investigations due to performing this method with computational methods. Also, the similarity between microbes and COVID-19 disease was studied cases, and 23 cases were confirmed using experimental articles. In the following, several case studies for microbes have been found to be obtained using similarities, and AFG has an effect on them and is confirmed by clinical results.

### Validation analytical and experimental results

This study investigates and substantiates the disease-microbe-drug relationship. We validated our findings by comparing them with selected databases and published articles. The collection of microbe-disease and microbe-drug relationship databases and articles was used to prove the existing relationships.

For instance, the relationship between AFG and the A. niger was investigated. According to the literature and databases, A. niger infections are relatively rare and usually occur fatally in immunosuppressed patients^77,78^. Voriconazole is the first-line treatment for invasive aspergillosis; however, A. niger has shown in vitro resistance to azoles, and no successful treatment cases with voriconazole have been reported^79,80^.Both methods used in this study demonstrated the efficacy of AFG against A. niger. In summary, our findings align with experimental data supporting the efficacy of AFG against A. niger, highlighting its potential for treating invasive aspergillosis^76^.

Then, the relationship between A. niger microbe and COVID-19 disease was investigated, and the results showed that patients with COVID-19 had significant changes in their fecal mycobiome (fecal fungal microbiomes) compared to the control group, with enrichment of Candida albicans and a very heterogeneous mycobiome configuration at the time of hospitalization. The diversity of fecal mycobiome in the last sample collected from patients with COVID-19 was 2.5 times higher than that of the control group (*P* < 0.05). Moreover, A. niger was detected in stool samples from some patients with COVID-19, even after clearance of SARS-CoV-2 from nasopharyngeal samples and resolution of respiratory symptoms^81^.

In addition, Suramin, penciclovir, and AFG were found to bind to nsp12 with similar binding energies as that of remdesivir, which has been used as a therapy for COVID-19. Recent experimental evidence also indicated that these drugs exhibit antiviral efficacy against SARS-CoV-2^82^.

Table 3 demonstrated some cases of triple drug-microbe-disease connections which were confirmed by experimental data and laboratory articles. More examples of these connections can be seen in the supplementary file. Due to the absence of the source or conducting of experimental tests, the remaining cases require further research. Case studies for type 2 diabetes (T2D), bipolar disorder, and autism demonstrate the favorable effectiveness of TNRGCN in association prediction^83^.

**Table 3:** The relationships between drug-microbe-disease connections obtained from the similarity and the sources found between them.

Drug	Microbe	Disease	Related references
AFG	A. fumigatus	COVID-19	PMID: 34784490
	Candida parapsilosis	COVID-19	PMID: 34784490
	Dengue Virus Type 2	COVID-19	PMID: 34784490
Ciprofloxacin	Staphylococcus aureus	Asthma	PMID: 39959969 PMID:36168550
	Staphylococcus aureus	Colorectal carcinoma	PMID: 40643531 PMID:11875713
	Staphylococcus aureus	COPD	PMID: 32267724 PMID: 20536364
	Haemophilus	Colorectal carcinoma	PMID: 11875713 PMID:40643531
	Haemophilus	Bowel disease	PMID: 36774550 PMID: 36144470
	Escherichia coli	IBD	PMID: 40512677 PMID:21853049
	Haemophilus	IBD	PMID: 36144470
Anidulafungin	A. fumigatus	CAPA	PMID: 33333012
Sorivudine	Bacteroides spp	Toxicity	PMID: 9690942
Metformin	A. muciniphila, etc.	T2DM	PMID: 28434033
SER-109 / FMT	Commensal flora	rCDI	PMID: 35045228
Risperidone	SCFA-related taxa	Obesity	PMID: 31215494

AFG, Anidulafungin; A.fumigatus, Aspergillus fumigatus; IBD, Inflammatory bowel disease; COPD, Chronic obstructive pulmonary disease; rcdi, Recurrent Clostridioides difficile Infection.

A comparison of the computational model with a number of representative method techniques from the recent literature is shown in Table 4. This article innovatively combines ternary links between microorganisms, drugs, and diseases into a single heterogeneous network, whereas most current models focus on binary relationships, usually microbe-drug or microbe-disease. Our approach explores significant biological information such as symptom overlap, microbe-functional profiles, and drug-structure similarity, in contrast to previous research that mainly relies on structural similarity or network topology alone. Furthermore, multiple real-world case studies and literature-verified connections are used to validate the proposed approach and enhance its therapeutic applicability. On the other hand, they are not empirically validated or only cover smaller parts of the biological interaction space.

Regarding interactions, unlike other studies that have only addressed dual interactions such as microbe-drug^50^, drug-microbe^60^, or drug-microbiome^51^, our study reported and evaluated triple interactions between microbe, drug, and disease. Regarding the integration of biological information, this paper presents a complete biological integration using clinical symptoms structural and functional similarity. While Tan et al.^50^ and Long et al.^60^ are limited to limited network or structural features, and Forslund et al.^51^ has no predictive component.

The predictions of this study are supported by case studies and evidence from the scientific literature. In contrast, Tan et al.^50^ only predicted microbe–drug interactions, Long et al.^60^ had limited predictions for drug–microbe interactions, and Forslund et al.^51^ had no predictions at all. We also validated the findings by reviewing the scientific literature (e.g., AFG–A. fumigatus–COVID-19 interactions). Tan, Long, and Gong had limited or no validation, and Forslund focused more on observational hospital data.Unlike other papers that lack case studies, we analyzed real-world disease examples such as type 1 diabetes, inflammatory bowel disease (IBD), COVID-19, autism, and bipolar disorder. Forslund only looked at the impact of drugs on the microbiome, and Tan and Long did not provide any case studies.

**Table 4:** Comparing study with a number of sample method techniques from the literature.

Features / Study	Study (Shokri Garjan et al., 2024)	Tan et al.^50^ 2022	Long et al.^60^ 2020	Gong et al.^46^ 2022	Forslund et al.^51^ 2021
Interaction Type	Microbe–Drug–Disease (Tripartite)	Microbe–Drug	Drug–Microbe	Microbe–Disease	Drug–Microbiome
Data Sources	HMDAD, Disbiome, MDAD	DrugBank, MDAD	DrugBank, genomic databases	HMDAD + heterogeneous networks	Clinical human data only
Model Type	Cytoscape + Random Walk + Gaussian similarity integration	Graph Attention Network + Sparse Autoencoder	Graph Convolutional Network + Conditional Random Field	Graph Neural Network with multi-source integration	Statistical descriptive analysis
Biological Integration	Symptoms, structural similarity, functional similarity	Limited biological integration	Structure-based only	Limited to network features	Real-world biological observations
Experimental Validation	Literature and lab-based validation (e.g., AFG–A. fumigatus–COVID-19)	Limited or absent	Indirect	Indirect	Based on hospital cohort data
Case Studies	Type 1 Diabetes, IBD, COVID-19, Autism, Bipolar Disorder	None reported	None reported	None reported	Drug effects on microbiome

## Discussion and conclusion

The microbiota plays a significant role in influencing the health and disease of individuals. Beyond its function in elucidating disease mechanisms, it can serve as a biomarker for both diagnosing and predicting diseases^91,92^. Disturbance of the body’s microbiome, induced either by drug treatment or alterations in living conditions like diet and exercise, can ameliorate disease^93^. Furthermore, the transplantation of microbiomes from healthy donors is emerging as an approach to disease therapy^94^. In this study, disease similarity was computed based on microbiota from all examined organs. Despite the predominant focus on microbiota from a single organ, primarily the gut, this has only a minor impact on the calculation of disease similarity. The microbe-based disease network remains small. Therefore, the existing associations between microorganisms and diseases encompass only a limited fraction of the vast array of microorganisms and human diseases. Recently, microbiomes have garnered increasing attention from medical researchers. It is expected that by reducing the cost of sequencing, more microbiomes of human diseases will be identified, and subsequently, a larger network of microbe-diseases and microbe-drugs will be built.

Currently, the global health landscape is grappling with the pandemic induced by the SARS-CoV-2 virus, necessitating an urgent quest for effective drugs for treatment and prevention. Given the substantial influence of human microbes on human health, research exploring the associations between microbes and drugs has garnered heightened attention. Accurately predicting potential connections between microorganisms and drugs can advance the development of targeted drugs and enhance our understanding of the intricate relationship between microorganisms and drugs. However, the majority of discoveries regarding associations between microbes and drugs are achieved through biological experiments, which are both time-consuming and costly. Therefore, the development of an effective computational method for the prediction of microbe-drug associations is necessary^95^.

In human health, drugs, germs, and disease are inherently linked.Building an extensive drug-microbe-disease network and archive for human microbial systems is therefore very desired. further looked closely at the different relationships between medications, microorganisms, and illnesses by locating trustworthy empirical sources. The likelihood of a disease-microbe relationship in score function-based models has the benefit of having a very simple algorithm theory and computational procedure. When these models are utilized for prediction, negative samples—which are extremely difficult to obtain—are not required when using the depth-first search technique to explore all of the connecting routes of microbial nodes and disease nodes in the heterogeneous network.

We examined the relationships between the microbe Staphylococcus aureus and asthma and colorectal carcinoma, as well as the aforementioned microbe and the drug Ciprofloxacin, which were shown to be related by reliable empirical databases and articles. Then, considering that the dual relationships were valid, we followed up by confirming the triple relationship between the disease, microbe, and drug mentioned. Reliable databases and articles also confirmed our findings. However, due to the lack of research on the relationships between the drug-microbe or disease-microbe, some results were not obtained from reliable sources. for Results from the largest registry study of antibiotics and asthma to date support the hypothesis that the association between antibiotic use and asthma may be due to methodological issues such as reverse causation or confounding by symptoms of respiratory tract infections. The researchers found that there was a significant association between asthma medication prescription and antibiotic use for Gram-positive infections, such as respiratory tract infections, but not for antibiotics for urinary tract or skin and soft tissue infections. Furthermore, the association was stronger for broad-spectrum antibiotics than for narrow-spectrum antibiotics^96^. For the drug ciprofloxacin and carcinoma, research results have shown Ciprofloxacin Mediated Cell Growth Inhibition, S/G2-M Cell Cycle arrest, and apoptosis in a human transitional cell carcinoma of the cyclin-dependent kinase inhibitor p21WAF1 level was found to be decreased within 12 h of ciprofloxacin treatment and disappeared completely when HTB9 cells were treated with 200 µg/ml ciprofloxacin for 24 h^97^.Nonetheless, there are very few disease-drug and microbe-drug interactions at play.

In further work, we will incorporate additional verified relationships as new datasets are established. Furthermore, the combining of multi-omics data may uncover additional biological information that aids in illness prediction, diagnosis, and therapy^98^ as a result of the thorough investigation of multi-omics such as transcriptomics^99^, proteomics^100^, and genomes^101^.

First off, the performance will be somewhat impacted by the sparse microbe-drug association matrix. Additionally, there are certain flaws in employing the microbe-disease relationship as an attribute characteristic because not all microorganisms (drugs) have illnesses linked to them. Lastly, to enhance the performance, we may add more biological data, such as interactions between microbes and between drugs. Furthermore, the framework is validated using real-world case studies (e.g., Anidulafungin–Aspergillus fumigatus–COVID-19, Metformin–gut microbiota–T2DM), and multiple predicted interactions are supported by experimental literature and biomedical databases. This empirical grounding strengthens its clinical relevance.

As microbiome research advances, it is expected to provide a more comprehensive understanding of disease–microbe–drug interactions. Improved outcomes may also be achieved by incorporating additional variables such as diet and probiotics. These tripartite relationships may serve as a foundation for future interventions targeting microbiome imbalances. By leveraging these relationships, researchers could potentially develop targeted therapies that either increase or decrease specific microbes to maintain or restore health. This unique contribution sets a new direction for subsequent research in the field and could lead to innovative treatments based on microbiome modulation. The primary aim of our study is to demonstrate that computational approaches can be used to systematically identify a set of candidate associations. To partially address validity, we attempted to support some of our findings by identifying experimental evidence reported in the literature. Nevertheless, we acknowledge that all predictions reported here should be further confirmed through direct experimental work (e.g., co-culture assays) by specialized research teams, which is beyond the scope of the present study.

While a limited fraction of samples is used in research, a large amount of data may be collected by using multi-omics approaches^102^. Stool microbiome characteristics only give a limited view of the lower gastrointestinal tract and cannot describe other gastrointestinal diseases in detail^103^. Retrospective comparisons of well-known datasets are the main foundation of clinical research; however, they might not be applicable in clinical situations^104^.

Different volumes of data have been needed to create, evaluate, and forecast the models, depending on the nature of machine learning algorithms. For instance, compared to neural networks and deep learning algorithms, linear regression and simple decision tree algorithms use a smaller selection of data. Therefore, it is necessary to enhance and broaden the present microbiome data sources^105^. The intricate relationship between disease, microorganisms (including pathogens and the microbiome), and medications is highlighted by the “disease-microbe-drug triad.” This framework has a number of drawbacks even though it is essential for comprehending disease causes and creating tailored treatments. Drug-microbiome interactions include the mutual impact on each other’s composition and function, the significant individual variability, and the difficulty of forecasting these interactions. Antimicrobial resistance results from the overuse of antibiotics, the existence of diverse resistance mechanisms, and its serious threat to global health. The complexity of the microbiome as a dynamic ecosystem with intertwined relationships and its intricate interactions with the host that are influenced by immunity and genetics. Limitations of computational modeling While computational models are useful for predicting interactions, they may struggle with sparse or noisy data. The future of the disease-microbe-drug interface requires a multifaceted approach that focuses on novel drug discovery, personalized therapies, and a deeper understanding of the role of the microbiome in disease and drug response. This includes investigating novel medication combinations, creating therapeutics based on the microbiome, and predicting drug efficacy and resistance using technologies like whole-genome sequencing and machine learning. Translating research findings into clinical practice also requires extensive bioinformatics knowledge and the integration of multi-omics (genomic, transcriptomic, etc.) data.

## Supplementary Information

Below is the link to the electronic supplementary material.

Supplementary Material 1

## Data Availability

The availability of data and content can be sent as a supplementary file in Excel format. Cytoscape software can be downloaded from the link [https://cytoscape.org](https:/cytoscape.org) .
